# Risk of COVID-19 in shielded and nursing care home patients: a cohort study in general practice

**DOI:** 10.3399/BJGPO.2021.0081

**Published:** 2021-10-27

**Authors:** David Wingfield, Mansour Taghavi Azar Sharabiani, Azeem Majeed, Mariam Molokhia

**Affiliations:** 1 Department of Primary Care and Public Health, Imperial College London, London, UK; 2 Department of Metabolism, Digestion and Reproduction, Imperial College London, London, UK; 3 School of Population Health and Environmental, King’s College London, London, UK

**Keywords:** COVID-19, ethnic groups, shielding, nursing homes, general practice, primary healthcare

## Abstract

**Background:**

COVID-19 cases were first detected in the UK in January 2020 and vulnerable patients were asked to shield from March to reduce their risk of COVID-19 infection.

**Aim:**

To determine the risk and determinants of COVID-19 diagnosis in shielded versus non-shielded groups, adjusted for key comorbidities not explained by shielding.

**Design & setting:**

Retrospective cohort study of adults with COVID-19 infection between 1 February 2020 and 15 May 2020 in west London.

**Method:**

Individuals diagnosed with COVID-19 were identified in SystmOne records using clinical codes. Infection risks were adjusted for sociodemographic factors, nursing home status, and comorbidities.

**Results:**

Of 57 713 adults, 573 (1%) individuals were identified as shielded and 1074 adults had documented COVID-19 infections (1.9%). COVID-19 infection rate in the shielded group individuals compared with non-shielded adult individuals was 6.5% (*n* = 37/573) versus 1.8% (*n* = 1037/57 140), *P*<0.001. A multivariable fully adjusted Cox proportional hazards (CPH) regression identified that COVID-19 infection was increased with shielding status (adjusted hazard ratio [aHR] 1.52; 95% confidence interval [CI] = 1.00 to 2.30; *P* = 0.048). Other determinants of COVID-19 infection included nursing home residency (aHR 7.05; 95% CI = 4.22 to 11.77; *P*<0.001); Black African (aHR 2.52; 95% CI = 1.99 to 3.18; *P*<0.001), Other (aHR 1.74; 95% CI = 1.42 to 2.13*; P*<0.001), Non-stated (aHR 1.70; 95% CI = 1.02 to 2.84; *P* = 0.04), or South Asian ethnic group (aHR 1.46; 95% CI = 1.10 to 1.93; *P* = 0.01); history of respiratory disease (aHR 1.51; 95% CI = 1.06 to 2.16; *P* = 0.02); deprivation (third versus least deprived Index of Multiple Deprivation [IMD] quintile) (aHR 1.25 ; 95% CI = 1.01 to 1.56; *P* = 0.05); obesity (body mass index [BMI] >30 kg/m^2^) (aHR 1.39; 95% CI = 1.18 to 1.63; *P*<0.001); and age (aHR 1.02; 95% CI = 1.01 to 1.02; *P*<0.001. Male sex was associated with lower risk of COVID-19 infection (aHR 0.71; 95% CI = 0.62 to 0.82; *P*<0.001).

**Conclusion:**

Shielded individuals had a higher COVID-19 infection rate compared with non-shielded individuals, after adjusting for sociodemographic factors, nursing home status, and comorbidities.

## How this fits in

Shielding was introduced to protect individuals from COVID-19 infection risk. The study found health inequalities, with higher levels of COVID-19 in the shielded group compared with the non-shielded group, which persisted after adjusting for demographic factors, nursing home status, and comorbidities.

## Introduction

COVID-19 cases were first detected in the UK in January 2020.^1^ Capacity for testing for the virus was initially very restricted in the UK and was not widely available via NHS Test and Trace until May or June 2020. This meant that GPs initially largely identified cases during clinical consultations using the following key COVID-19 diagnostic symptoms: cough, fever, breathlessness, and loss (or change) of the sense of smell. The NHS was rapidly supplied with a set of new codes and templates for GPs to record symptoms, physical signs, and diagnoses. Patients accessed the NHS through all available routes, including GPs, NHS 111, the ambulance service, and hospital accident and emergency (A&E) departments, and the distribution of such presentations is likely to have reflected severity as well as patient concern.

Early in the pandemic, it was recognised that certain patients were at particularly high risk owing to their concurrent medical illnesses.^2–4^ This was the basis for a programme to protect high-risk individuals and was defined in a letter from the Chief Medical Officer for England^5^ on 23 March 2020. This required GPs and hospital specialist units to contact their patients by letter and phone call to alert them of the need to protect themselves (Box 1). Government letters were also sent to patients. The programme involved asking this ‘shielded’ cohort of patients to stay at home, being supplied with essential items via local authority action and voluntary agencies, according to shielding list procedure.^6^ The programme started on 23 March but was expanded from an initial target of 1.5 million patients to more than double this number in three subsequent cohorts.^7^ The aim was to ensure that shielded patients received minimal exposure to SARS-CoV-2, thereby reducing the infection rate and subsequent morbidity and mortality.

Box 1 **First shielding criteria March 2020 (withdrawn 1 May 2020;^11^ now updated^24^)**

We are advising those who are at increased risk of severe illness from coronavirus (COVID-19) to be particularly stringent in following social distancing measures.

This group includes those who are:

- aged 70 or older (regardless of medical conditions)
- under 70 with an underlying health condition listed below (ie anyone instructed to get a flu jab as an adult each year on medical grounds):
  - chronic (long-term) mild-to-moderate respiratory diseases such as asthma, chronic obstructive pulmonary disease (COPD), emphysema, or bronchitis
  - chronic heart disease such as heart failure
  - chronic kidney disease
  - chronic liver disease such as hepatitis
  - chronic neurological conditions such as Parkinson’s disease, motor neurone disease, multiple sclerosis (MS), a learning disability or cerebral palsy
  - diabetes
  - a weakened immune system as the result of conditions such as HIV and AIDS, or medicines such as steroid tablets
  - being seriously overweight (a body mass index [BMI] of 40 or above)
- those who are pregnant

Note: there are some clinical conditions which put people at even higher risk of severe illness from COVID-19. If you are in this category, next week the NHS in England will directly contact you with advice about the more stringent measures you should take in order to keep yourself and others safe. For now, you should rigorously follow the social distancing advice in full, outlined below.

People falling into this group are those who may be at particular risk due to complex health problems such as:

- people who have received an organ transplant and remain on ongoing immunosuppression medication
- people with cancer who are undergoing active chemotherapy or radiotherapy
- people with cancers of the blood or bone marrow such as leukaemia who are at any stage of treatment
- people with severe chest conditions such as cystic fibrosis or severe asthma (requiring hospital admissions or courses of steroid tablets)
- people with severe diseases of body systems such as severe kidney disease (dialysis)

This study aimed to determine the risks of shielded patients (defined according to government guidance of 23 March 2020) acquiring COVID-19 infection in five general practices in west London compared with non-shielded adults, adjusted for nursing home status, demographic factors, and comorbidities.

## Method

### Study design

**Figure 1.**
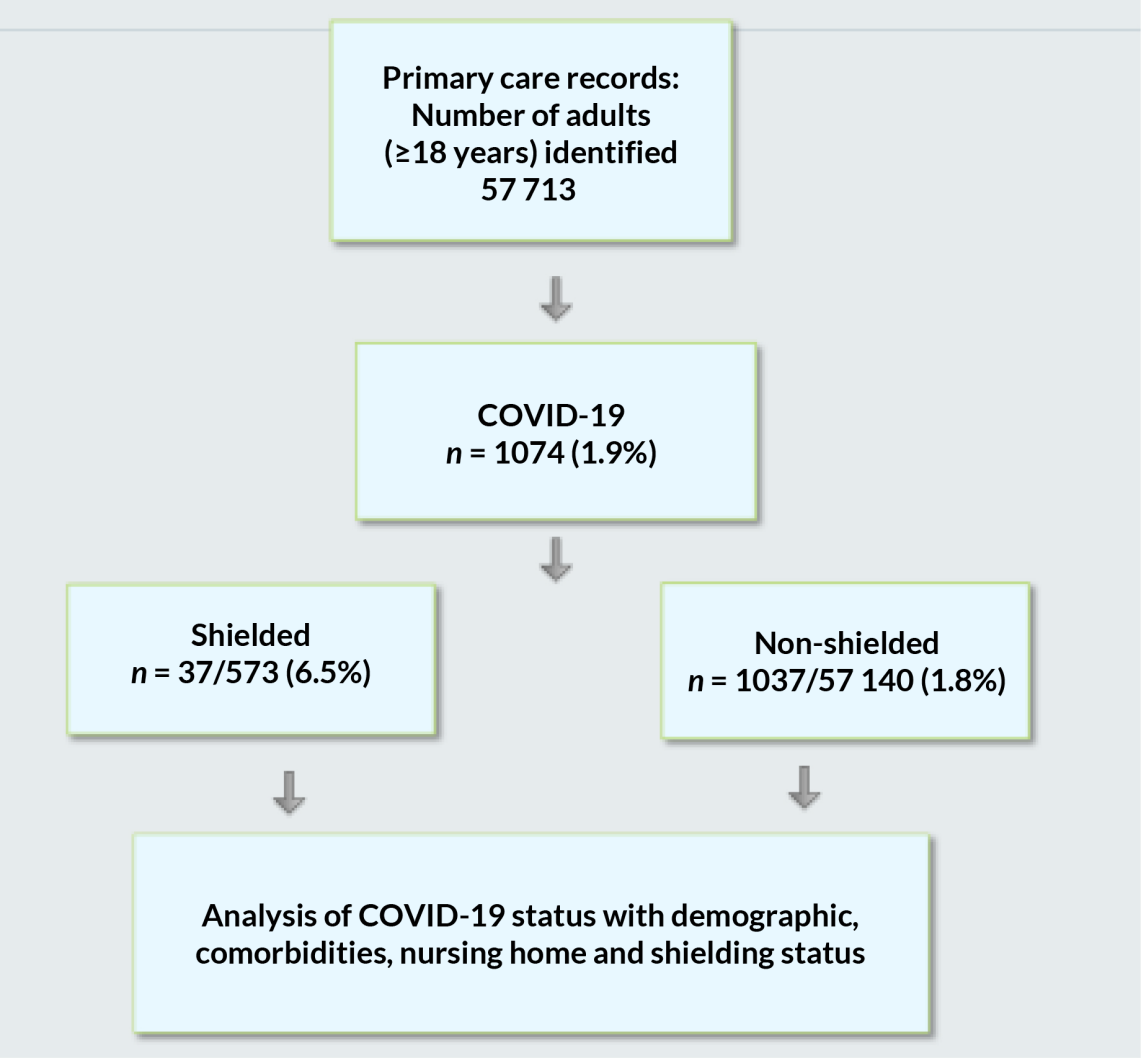
Study flowchart

A retrospective population-based cohort study using STROBE guidelines was conducted between 1 February 2020 and 15 May 2020, using a CPH model with people diagnosed with COVID-19 as the primary outcome adjusted for risk factors including shielded status. Individuals were censored when they were diagnosed with COVID-19, left the practice, or died. Details of the study selection are shown in Figure 1.

### Setting

Five general practices were studied in west London, UK, covering two nursing homes in Hammersmith and Fulham Clinical Commissioning Group.

### Data sources

A longitudinal study was undertaken in an ethnically diverse adult population, using primary care electronic health records (EHR) from five general practices (same partnership group). Patient level clinical data, prescribing data, laboratory data, and demographic information were examined, including ethnic group based on categories of the UK 2001 census, risk factors, and comorbidities. This was extracted from the SystmOne electronic clinical record. Shielding status, demographic and lifestyle factors, and comorbidities were investigated in a multi-ethnic population identified as having suspected or NHS laboratory confirmed COVID-19.

### Study population

The study was carried out using anonymised data from adult patients aged ≥18 years registered with five GP clinics in west London.

### Identification of COVID-19 status

COVID-19 status was determined using the COVID-19 diagnostic template based on clinical assessment for COVID-19 diagnosis (using current diagnostic guidelines) for the majority of cases, supplemented with NHS laboratory testing results where available. These codes were grouped together for combined analysis.

### Covariates (exposures)

Factors were examined such as age; sex; ethnic group; deprivation, (Index of Multiple Deprivation [IMD] 2019);^8^ BMI and selected comorbidities likely to affect health outcomes, including type two diabetes, hypertension (HTN), chronic kidney disease (CKD), coronary heart disease (CHD), and history of respiratory disease (chronic obstructive pulmonary disease [COPD] or asthma). Quality and Outcome Framework (QOF) registers were used at the time of the data extract and self-reported lifestyle factors such as smoking. Ethnic group was self-reported and aggregated into eight categories: White, Black African, South Asian, Chinese, Mixed, Other, Non-stated, and missing. It was also possible to identify by post code whether the patient resided in one of the two long-term nursing homes within the practice population.

### Outcomes

The following were examined:

1. Proportion of shielded group with COVID-19 infection.
2. Risk and determinants of receiving a shielded diagnosis.
3. Risk and determinants of receiving a COVID-19 diagnosis in shielded versus non-shielded groups, adjusted for key comorbidities not explained by shielding.

### Analysis

A multivariable multi-level logistic regression was used to assess factors associated with shielding status in adult individuals using Stata (version 16). Differences between proportions of categorical variables were assessed using a χ^2^ test. Predictors of shielding status were assessed by univariable and multivariable logistic regression (including missing categories), adjusted for practice effects and other confounders.

A CPH model adjusted for practice examined the association of demographic factors including deprivation, and comorbidities not associated with shielding, with COVID-19 infection status as an outcome. The proportional hazard assumptions were met, and log rank tests were used to assess significance. Partly adjusted (adjusted for age group and sex) and fully adjusted (adjusted for age group, sex, and other covariates) CPH analysis was conducted to adjust for potential confounders. The covariates adjusted for included ethnic group (White ethnic group as reference), nursing home residency, obesity (BMI >30 kg/m^2^), locally based IMD deprivation score quintile, smoking status, and comorbidities. Analyses included testing for interactions such as age group, sex, type two diabetes, and obesity in all models.

## Results

### Descriptive characteristics of the study population

The study population comprised 57 713 adults in five GP practices in west London. The mean duration of follow-up time was 102 days. The characteristics for individuals are summarised in Table 1 (by shielding status) and Table 2 (by COVID-19 infection status). Table 1 confirms that 6.5% of the shielded population were diagnosed with COVID-19 compared with 1.8% of the non-shielded population, and that this difference was highly significant (*P*<0.001). The shielded patient group contains significantly more female, older, and Black African patients, with higher levels of comorbidities (additional to those related to shielding), higher BMI, and greater deprivation. It was therefore important to adjust for these differences in determining whether shielded patients had higher infection rates or not.

**Table 1.** Summary characteristics of shielded (7 April 2020) and non-shielded in 57 713 adults aged ≥18 years

**Variables**	**Shielded** *n* = 573	**Non-shielded** *n* = **57 140**	*P* value
***n***	**Col %**	*n*	**Col %**
COVID-19 infection		37	6.5	1037	1.8	<0.001
Sex	Female	341	59.5	28 974	50.7	<0.001
	Male	232	40.5	28 164	49.3	
	Missing	0	0	0	0	
Age, years	<30	32	5.6	11 512	20.2	<0.001
	30–39	48	8.4	16 945	29.7	
	40–49	53	9.3	11 389	19.9	
	50–59	111	19.4	8207	14.4	
	60–69	118	20.6	4646	8.1	
	70–79	142	24.8	2848	5.0	
	≥80	69	12.0	1593	2.8	
	Nursing home resident	8	1.4	178	0.3	<0.001
BMI (kg/m^2^)	Underweight (<18.5 kg/m^2^)	26	4.1	2350	4.5	<0.001
	Normal weight(18.5–24.9 kg/m^2^)	169	29.5	20 981	36.7	
	Pre-obesity(25.0–29.9 kg/m^2^)	122	21.3	11 074	19.4	
	Obese I(30.0–34.9 kg/m^2^)	78	13.6	3855	6.8	
	Obese II(35.0–39.9 kg/m^2^)	22	3.8	1291	2.3	
	Obese III(≥40 kg/m^2^)	17	3.0	714	1.3	
	Missing value	139	24.3	16 875	29.5	
IMD quintile (% coded)	1 (most deprived)	145	25.3	10 945	19.2	0.009
	2	107	18.7	10 935	19.1	
	3	114	19.9	11 565	20.2	
	4	95	16.6	11 297	19.8	
	5 (least deprived)	95	16.6	10 502	18.4	
	Missing	17	3.0	1896	3.3	
Ethnic group	White	157	27.4	18 294	32.0	<0.001
	Black African	71	12.4	3537	6.2	
	Chinese	1	0.2	1067	1.9	
	Asian	34	5.9	3654	6.4	
	Mixed	170	29.7	16 080	28.1	
	Other	101	17.6	7724	13.5	
	Non-stated	12	2.1	727	1.3	
	Missing	27	4.7	6057	10.6	
History of comorbidities	Type 2 diabetes	112	19.6	2647	4.6	<0.001
	HTN	153	26.7	4042	7.1	<0.001
	CKD	91	15.9	1198	2.1	<0.001
	CHD	62	10.8	973	1.7	<0.001
	Respiratory disease	178	31.1	691	1.2	<0.001
Lifestyle factors	Does not smoke	218	38.1	32 897	57.6	<0.001
	Currently smokes	130	22.7	10 208	17.9	
	Formerly smoked	221	38.6	11 596	20.3	
	Missing	4	0.7	2439	4.3	

CHD = coronary heart disease. Col = column. CKD = chronic kidney disease. HTN = hypertension. IMD = Index of Multiple Deprivation.

**Table 2.** Summary characteristics of adults infected with COVID-19 compared with those not infected in 57 713 adults aged ≥18 years

	**COVID-19** ***n* = 1074** (**1.9%**)	**No COVID-19 infection** ***n* = 56 639** (**98.1%**)	***P* value**
Sex			
Female	651 (60.6)	28 664 (50.6)	<0.001
Male	423 (39.4)	27 973 (49.4)	
Age, years			<0.001
18–30	94 (8.8)	11 450 (20.2)	
30–39	202 (18.8)	16 791 (29.7)	
40–49	223 (20.8)	11 219 (19.8)	
50–59	258 (24.0)	8060 (14.2)	
60–69	137 (12.8)	4627 (8.2)	
70–79	84 (7.8)	2906 (5.1)	
≥80	76 (7.1)	1586 (2.8)	
Ethnic group *n* (%)			
White	251 (23.4)	18 200 (32.1)	<0.001
South Asian	81 (7.5)	3607 (6.4)	
Black African	138 (12.9)	3470 (6.1)	
Chinese	11 (1.0)	1057 (1.9)	
Mixed	268 (25.0)	15 982 (28.2)	
Other	221 (20.6)	7604 (13.4)	
Non-stated	23 (2.1)	716 (1.3)	
Missing	81 (7.5)	6003 (10.6)	
Lifestyle indicators			
Nursing home resident	52 (4.8)	134 (0.2)	<0.001
Not obese	794 (73.9)	44 437 (78.5)	<0.001
Obese (BMI >30 kg/m^2^)	230 (21.4)	7112 (12.6)	
Missing	50 (4.7)	5090 (9.0)	
IMD quintile (% coded)			
1 (most deprived)	241 (22.4)	10 849 (19.2)	<0.001
2	233 (21.7)	10 809 (19.1)	
3	219 (20.4)	11 460 (20.2)	
4	176 (16.4)	11 216 (19.8)	
5 (least deprived)	156 (14.5)	10 441 (18.4)	
Missing	49 (4.5)	1864 (3.3)	
Smoking status			<0.001
Currently smokes	174 (16.2)	10 164 (18.0)	
Formerly smoked	265 (24.7)	11 552 (20.4)	
Does not smoke	623 (58.0)	32 492 (57.4)	
Missing	12 (1.1)	2431 (4.3)	
Comorbidities			
Type 2 diabetes	128 (11.9)	2631(4.7)	<0.001
HTN	153 (14.3)	4042 (7.1)	<0.001
CKD	57 (5.3)	1232 (2.2)	<0.001
CHD	48 (4.5)	987 (1.7)	<0.001
Respiratory disease	54 (5.0)	815 (1.4)	<0.001

CHD = coronary heart disease. CKD = chronic kidney disease. HTN = hypertension. IMD = Index of Multiple Deprivation.

### Determinants of receiving a ‘shielded’ diagnosis

In the partially adjusted (adjusted for age and sex) logistic regression (Table 3), the following were associated with an increased odds of shielding:

- History of respiratory disease (adjusted odds ratio [aOR] 15.16; 95% CI = 12.24 to 18.76; *P*<0.001)
- Smoking (aOR 2.45; 95% CI = 1.96 to 3.07), and ex-smoking status (aOR 2.16; 95% CI = 1.78 to 2.62), both *P*<0.001
- Black African ethnic group (aOR 2.23; 95% CI = 1.68 to 2.97; *P*<0.001)
- CKD (aOR 2.05 ; 95% CI = 1.58 to 2.67; *P*<0.001)
- CHD (aOR 1.97; 95% CI = 1.47 to 2.63; *P*<0.001)
- Type 2 diabetes (aOR 1.95 ; 95% CI = 1.56 to 2.45; *P*<0.001)
- Obesity (aOR 1.60; 95% CI = 1.32 to 1.94; *P*<0.001)
- HTN (aOR 1.45; 95% CI = 1.17 to 1.79; *P* = 0.001)
- Age (aOR 1.06; 95% CI = 1.05 to 1.06; *P*<0.001) and
- IMD level 5 compared with least deprived (aOR 1.48; 95% CI =1.14 to 1.92; *P* = 0.003).

**Table 3.** Partially and fully adjusted multi-level mixed effects regression of the odds of shielding status in 57 713 adults aged ≥18 years

**Shielding status**	**Partially adjusted odds ratio 95%** CI^a^	***P* value**	**Fully **adjusted**** **odds ratio 95%** CI^b^	***P* value**
Age (years)	1.06 (1.05 to 1.06)	<0.001	1.03 (1.03 to 1.04)	<0.001
Sex				
Female	ref		ref	
Male	0.74 (0.63 to 0.88)	<0.001	0.62 (0.52 to 0.75)	<0.001
Nursing home resident	0.80 (0.39 to 1.65)	0.54	1.07 (0.35 to 3.33)	0.90
BMI				
BMI <30 kg/m^2^	ref		ref	
Obese >30 kg/m^2^	1.60 (1.32 to 1.94)	<0.001	1.32 (1.07 to 1.64)	0.01
Smoking status				
Does not smoke	ref		ref	
Currently smokes	2.45 (1.96 to 3.07)	<0.001	1.50 (1.16 to 1.94)	<0.001
Formerly smoked	2.16 (1.78 to 2.62)	<0.001	1.68 (1.35 to 2.10)	<0.001
Ethnic group				
White	ref		ref	
South Asian	0.98 (0.67 to 1.42)	0.91	1.37 (0.92 to 2.03)	0.12
Black African	2.23 (1.68 to 2.97)	<0.001	2.78 (2.02 to 3.81)	<0.001
Chinese	0.15 (0.02 to 1.07)	0.06	0.23 (0.03 to 1.64)	0.14
Mixed	1.11 (0.89 to 1.38)	0.36	1.11 (0.87 to 1.41)	0.40
Other	1.13 (0.88 to 1.46)	0.33	1.26 (0.95 to 1.67)	0.11
Non-stated	1.80 (0.99 to 3.28)	0.06	2.23 (1.16 to 4.27)	0.02
Missing	0.50 (0.33 to 0.75)	0.001	0.84 (0.54 to 1.30)	0.42
IMD quintile (% coded)				
1 (least deprived)	ref			
2	0.94 (0.71 to 1.25)	0.68	0.98 (0.72 to 1.33)	0.90
3	1.10 (0.83 to 1.44)	0.50	1.16 (0.86 to 1.55)	0.33
4	1.09 (0.83 to 1.44)	0.53	0.97 (0.72 to 1.31)	0.85
5 (most deprived)	1.48 (1.14 to 1.92)	0.003	1.24 (0.93 to 1.64)	0.14
Comorbidities				
Type 2 diabetes	1.95 (1.56 to 2.45)	<0.001	1.39 (1.07 to 1.80)	0.01
HTN	1.45 (1.17 to 1.79)	0.001	1.07 (0.84 to 1.36)	0.58
CKD	2.05 (1.58 to 2.67)	<0.001	1.96 (1.47 to 2.63)	<0.001
CHD	1.97 (1.47 to 2.63)	<0.001	1.21 (0.87 to 1.68)	0.27
Respiratory disease	15.16 (12.24 to 18.76)	<0.001	12.72 (10.00 to 16.18)	<0.001

BMI = body mass index; IMD = Index of Multiple Deprivation; HTN = hypertension; CKD = chronic kidney disease; CHD = coronary heart disease

a Adjusted for age and sex.

b Adjusted for all covariates in the table and practice.

Male sex was associated with decreased odds of shielding (aOR 0.74; 95% CI = 0.63 to 0.88; P<0.001).

In the fully adjusted logistic analyses, the following were associated with increased odds of shielding:

- History of respiratory disease (odds ratio [OR] 12.72; 95% CI = 10.00 to 16.18; *P*<0.001)
- Black African (OR 2.78; 95% CI = 2.02 to 3.81; *P*<0.001), and Non-stated ethnic group (OR 2.23; 95% CI = 1.16 to 4.27; *P* = 0.02)
- CKD (OR 1.96; 95% CI = 1.47 to 2.63; *P*<0.001)
- Smoking (OR 1.50; 95% CI = 1.16 to 1.94; *P*<0.001) and ex-smoking status (OR 1.68; 95% CI = 1.35 to 2.10; *P*<0.001)
- Type 2 diabetes (OR 1.39; 95% CI = 1.07 to 1.80; *P* = 0.01)
- Obesity (OR 1.32; 95% CI = 1.07 to 1.64; *P* = 0.01)
- Older age (OR 1.03; 95% CI = 1.03 to 1.04; *P*<0.001).

Male sex was associated with decreased odds of shielding (OR 0.62; 95% CI = 0.52 to 0.75; *P*<0.001).

### Characteristics of the COVID-19 infections

**Figure 2.**
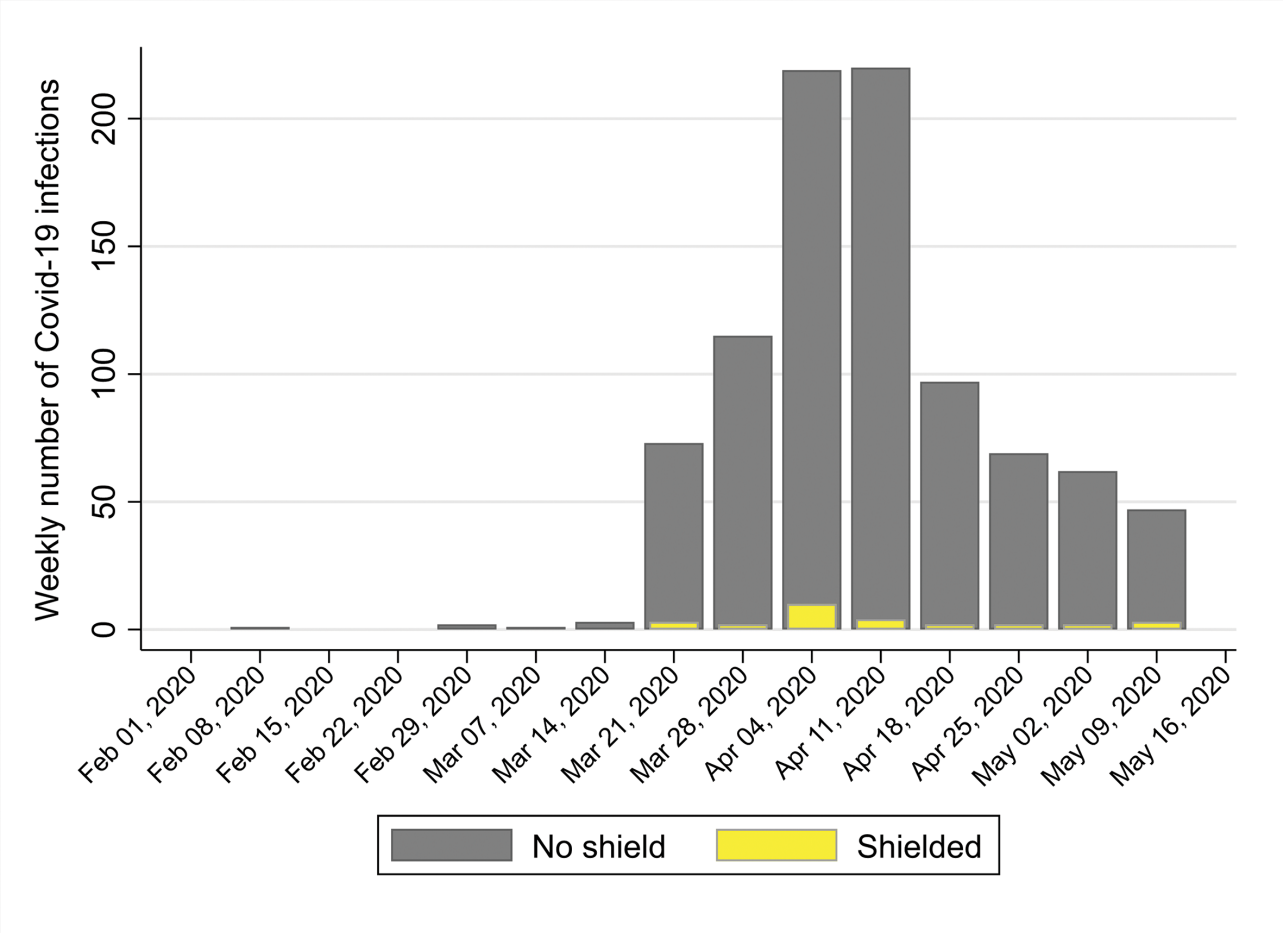
Weekly cases of COVID-19 infections by shielding categories from 23 March 2020 to 15 May 2020

Figure 2 shows the incident cases per week during the study period and confirms that peak incidence was in the weeks of 4 April and 11 April 2020. There were *n* = 3/28 cases in shielded patients occurring before 28 March 2020, the first full week of shielding (10.7%). Table 2 shows older age, nursing home residence, Black African or South Asian ethnic group, obesity, CKD, HTN, CHD, respiratory illness, and type 2 diabetes were all significantly higher in those with COVID-19. Figure 2, which shows weekly COVID-19 cases by shielding status, suggests some shielded individuals may not have been adequately shielded, or experienced household contacts and/or other exposure.

### Determinants of COVID-19 infection

All reported analyses cover the full study period 1 February 2020 to 15 May 2020. In the partially adjusted CPH analyses, adjusted for age and sex, the following were associated with increased risk of COVID-19 infection:

- Nursing home residency (aHR 9.37; 95% CI = 6.68 to 13.15; *P*<0.001)
- Shielded status (aHR 2.00; 95% CI = 1.35 to 2.86; *P*<0.001)
- Black African (aHR 2.68; 95% CI = 2.16 to 3.34; *P*<0.001), Non-stated (aHR 2.10; 95% CI = 1.34 to 3.30; *P* = 0.001), Other (aHR 1.75; 95% CI = 1.45 to 2.12; *P*<0.001) or South Asian ethnic group (aHR 1.48; 95% CI = 1.14 to 1.93; *P* = 0.004)
- Obesity (aHR 1.50; 95% CI = 1.29 to 1.76; *P*<0.001)
- Third (aHR 1.33; 95% CI = 1.07 to 1.64; *P* = 0.01), fourth (aHR 1.31; 95% CI = 1.05 to 1.63; *P* = 0.02), and fifth deprivation quintiles (aHR 1.46; 95% CI = 1.17 to 1.81; *P* = 0.001)
- Comorbidities: type 2 diabetes (aHR 1.63; 95% CI = 1.31to 2.02; *P*<0.001), HTN (aHR 1.25; 95% CI = 1.02 to 1.53; *P* = 0.03), CHD (aHR 1.38; 95% CI = 1.00 to 1.90; *P* = 0.01), and history of respiratory disease (aHR 1.68; 95% CI = 1.23 to 2.31; *P*<0.001).

Male sex was associated with lower risk of COVID-19 infection (aHR 0.68; 95% CI = 0.60 to 0.77; *P*<0.001) Table 4.

**Table 4.** Partially and fully adjusted Cox proportional hazards regression of the odds of COVID-19; during 1 February 2020–15 May 2020 in 57 713 adults aged ≥18 years

**COVID-19 infection**	**Partially adjusted** **hazard ratio 95%** CI^a^		**Fully adjusted hazard ratio 95%** CI^b^	**Fully adjusted *P* value**
Age, years	1.03 (1.02 to 1.03)	<0.001	1.02 (1.01 to 1.02)	<0.001
Sex				
Female	–		–	
Male	0.68 (0.60 to 0.77)	<0.001	0.71 (0.62 to 0.82)	<0.001
Shielded group	2.00 (1.35 to 2.86)	<0.001	1.52 (1.00 to 2.30)	0.048
Nursing home resident	9.37 (6.68 to 13.15)	<0.001	7.05 (4.22 to 11.77)	<0.001
BMI				
BMI <30 kg/m^2^	ref		ref	
Obese >30 kg/m^2^	1.50 (1.29 to 1.76)	<0.001	1.39 (1.18 to 1.63)	<0.001
Smoking status				
Does not smoke	ref		ref	
Currently smokes	0.90 (0.75 to 1.08)	0.24	0.90 (0.74 to 1.09)	0.29
Formerly smoked	1.02 (0.87 to 1.19)	0.81	1.10 (0.94 to 1.30)	0.24
Ethnic group				
White	ref		ref	
South Asian	1.48 (1.14 to 1.93)	0.004	1.46 (1.10 to 1.93)	0.01
Black African	2.68 (2.16 to 3.34)	<0.001	2.52 (1.99 to 3.18)	<0.001
Chinese	0.91 (0.50 to 1.66)	0.75	1.04 (0.57 to 1.91)	0.90
Mixed	1.11 (0.92 to 1.33)	0.28	1.12 (0.93 to 1.36)	0.24
Other	1.75 (1.45 to 2.12)	<0.001	1.74 (1.42 to 2.13)	<0.001
Non-stated	2.10 (1.34 to 3.30)	0.001	1.70 (1.02 to 2.84)	0.04
Missing	0.88 (0.67 to 1.16)	0.36	0.87 (0.64 to 1.18)	0.37
IMD quintile (% coded)				
1 (least deprived)	ref		ref	
2	1.13 (0.90 to 1.41)	0.30	1.07 (0.85 to 1.34)	0.58
3	1.33 (1.07 to 1.64)	0.01	1.25 (1.01 to 1.56)	0.05
4	1.31 (1.05 to 1.63)	0.02	1.11 (0.88 to 1.40)	0.38
5 (most deprived)	1.46 (1.17 to 1.81)	0.001	1.21 (0.97 to 1.53)	0.10
Comorbidities				
Type 2 diabetes	1.63 (1.31 to 2.02)	<0.001	1.26 (0.99 to 1.60)	0.06
HTN	1.25 (1.02 to 1.53)	0.03	1.01 (0.81 to 1.26)	0.93
CKD	1.00 (0.74 to 1.37)	0.98	0.79 (0.57 to 1.11)	0.17
CHD	1.38 (1.00 to 1.90)	0.01	1.20 (0.85 to 1.69)	0.29
Respiratory disease	1.68 (1.23 to 2.31)	<0.001	1.51 (1.06 to 2.16)	0.02

CHD = coronary heart disease. CKD = chronic kidney disease. HTN = hypertension. IMD = Index of Multiple Deprivation.

a Adjusted for age, sex, and practice.

b Adjusted for all covariates in the table and practice.

A multivariable fully adjusted CPH regression identified that COVID-19 infection was increased with: nursing home residency (aHR 7.05; 95% CI = 4.22 to 11.77; *P*<0.001), and shielding status (aHR 1.52; 95% CI = 1.00 to 2.30; *P* = 0.048).

Other determinants of COVID-19 infection found included:

- Black African (aHR 2.52; 95% CI = 1.99 to 3.18; *P*<0.001), Other (aHR 1.74; 95% CI = 1.42 to 2.13; *P*<0.001), Non-stated (aHR 1.70; 95% CI = 1.02 to 2.84; *P* = 0.04), or South Asian ethnic group (aHR 1.46; 95% CI = 1.10 to 1.93; *P* = 0.01)
- History of respiratory disease (aHR 1.51; 95% CI = 1.06 to 2.16; *P* = 0.02)
- Deprivation (third versus least deprived IMD quintile) (aHR 1.25; 95% CI = 1.01 to 1.56; *P* = 0.05)
- Obesity (BMI >30 kg/m^2^) (aHR 1.39; 95% CI = 1.18 to 1.63; *P*<0.001), and
- Age (aHR 1.02; 95% CI = 1.01 to 1.02; *P*<0.001).

Male sex was associated with lower risk of COVID-19 infection (aHR 0.71; 95% CI = 0.62 to 0.82; *P*<0.001). No statistical interaction in any of the models was found. Further details showing infection rate, which is more rapid in the shielded group and continues throughout the study period, attenuating by 15 May 2020 is given in the Kaplan–Meier plots and shielded numbers, see Supplementary materials (Figures S1–S3).

## Discussion

### Summary

Patients in the shielded group had a higher COVID-19 infection rate compared with non-shielded individuals, and this effect remained after adjusting for demographic factors, nursing home residence, confounders, and comorbidities. It was found nursing home status was a strong confounder of COVID-19 infection in the shielded patient cohort, and this is the first report able to distinguish the separate risk of these two vulnerable patient cohorts using a unique population. The shielded patient proportion of the nursing homes (*n* = 8/186; 4.3%) was higher than the shielded proportion in the non-nursing home population (*n* = 565/57 527; 0.98% *P*<0.001), with increased infection rates. This finding is consistent with previous reports in different patient cohorts that showed high mortality in these groups.^9,10^ During the first-wave peak, shielded cases mirror population COVID-19 infection numbers, suggesting some ongoing infection transmission via households or otherwise during the shielded period. The authors were unable to distinguish from their analysis whether the higher rate of diagnosis of COVID-19 in shielded patients was owing to a true higher incidence, or a greater level of symptom severity leading to a higher likelihood of presenting to primary care.

The study found that older age, obesity, type 2 diabetes, smoking, CKD, Black African and Non-stated ethnic group, and respiratory disease were associated with increased odds of shielding, and some comorbidities reflect shielding guidance.^11^ Male sex was associated with decreased odds of shielding. Patients with these common conditions will also have other comorbidities that are associated with shielding characteristics. However, the authors were unable to adjust fully for immunosuppressive medication, which is frequently prescribed from secondary care and immunosuppressive comorbidities may be under-recorded. It is noted that there were only three cases of infection before the implementation of the shielded patient scheme on 23 March 2020 in both shielded and non-shielded groups, and therefore this period has not been considered separately.

The study found other determinants of higher COVID-19 infection rates were age, Black African and South Asian ethnic groups, obesity (BMI >30 kg/m^2^), and history of respiratory disease, all consistent with previous reports.^3,12^ The fact that increased rates were found, but not reaching the level of significance for type 2 diabetes, CHD and hypertension, and CKD may reflect the sample size and lower power of the present study.

### Strengths and limitations

The study examined a number of risk factors for COVID-19 and the effect of shielding in a socioeconomically, ethnically diverse population in west London covering over 57 000 patients, and reflects individuals presenting with COVID-19 to general practice. Those seeking healthcare advice and support will therefore have had more severe symptoms excluding those with fulminant illness requiring immediate hospital transfer, and also those with mild or asymptomatic illness. The authors are also aware that GPs did not complete the templates in all cases and infection rates differed between the five practices, therefore practice was adjusted for in the models. No routine primary care testing was available between March and May 2020 during the study period, and therefore the majority of COVID-19-coded cases here are likely to be those clinically diagnosed via primary care telephone triage, which may be subject to misclassification. It is likely that shielded patients are more likely to be aware of such symptoms, experience overt symptoms (as opposed to asymptomatic or mild cases), report symptoms, and access health care (and possibly that GPs are also more proactive with diagnosing COVID-19 in this group). Data were not recorded on adherence to the shielding programme in this study and therefore this could not be reported.

Patients had multiple access points to the NHS; however, these GP practices had a clear denominator of registered patients, COVID-19 assessment and diagnostic centre, and were highly prominent with good telephone access during this time period. The epidemic curve and associations with demographic factors and pre-existing morbidity in the data are consistent with other studies.

The limitations apply to those found with observational data and include misclassification, missing data, and unmeasured confounders (such as frailty, and healthcare usage), including GP practice factors. As >98% of patients are registered with a GP, data capture is high. The effect and direction of bias could not be ascertained owing to missing data, introducing possible bias for BMI and CKD for non-coded versus coded patients.^13^ Other limitations include selection (owing to comorbidities and QOF coding) and survivor bias. In London, the population is younger and more deprived compared with the rest of the UK. Finally, the authors did not have access to complete hospital admissions data, or long-term outcomes such as mortality. The study may be underpowered to detect true effects of comorbidities owing to numbers of COVID-19 cases.

### Comparison with existing literature

The findings concur with that of Hull *et al* in a primary care population of 1.3 million,^14^ including an apparent protective risk for COVID-19 in men consulting in primary care. However, both of these studies are susceptible to collider bias,^15^ which may be owing to lack of mild or no symptoms and selection pressures bias samples toward those with decreased symptom severity and lower numbers of men consulting in primary care. Jani *et al* also found excess risk of COVID-19 infection in shielded individuals in a general population cohort, but did not adjust for NH residency.^16^

The fully adjusted model includes deprivation and reveals that the findings are significant in spite of deprivation status, indicating a biological underlying factor. COVID-19 is a new illness and much about it remains to be discovered. However, it is already known that it involves a hyperactivation of the immune and clotting systems of the body and that this can be ameliorated by the administration of dexamethasone.^17^ Recent trials have indicated that treatment with the interleukin-6 (IL-6) receptor antagonists, tocilizumab and sarilumab, may improve outcome, including survival in critically ill patients with COVID-19 in intensive care.^18^ Diabetes and obesity are associated with altered immune states,^19^ as is advancing age, which may render individuals more susceptible to COVID-19 infection and mortality.^20^ Multimorbidity and physical frailty may additionally be independent risk factors in this illness; this study did not assess these independently.

### Implications for research and practice

Patients in the shielded group have a higher COVID-19 infection rate compared with non-shielded individuals, after adjusting for demographic factors, confounders, and comorbidities. This suggests that shielded patients, along with nursing home patients, were more exposed to COVID-19 infection than public policy intended,^21^ and that exiting lockdown strategies should take this into account.^22,23^ The results suggest that shielding alone is not enough to protect vulnerable people and that ongoing vaccination programmes remain the best way to protect these patient groups from the risk of serious illness and death from COVID-19. It is expected that shielded patients are more likely to experience symptomatic COVID-19 (which were largely the infections that were detectable during the study period, owing to minimal testing availability), which would have been coded by GPs. This may inform future community-shielding strategies and management in primary care, for future COVID-19 waves and research.

Demographic factors associated with COVID-19 were nursing home residency; shielded status; Black African, South Asian, Other, or Non-stated ethnic group; obesity; type 2 diabetes; and age. The association with Black African, South Asian, and Other ethnic group is important as it demonstrates an ethnic health inequality, which remained after adjusting for deprivation, as mortality rates in COVID-19 are increased in these groups.
